# Stimulation induces gradual increases in the thickness and curvature of postsynaptic density of hippocampal CA1 neurons in slice cultures

**DOI:** 10.1186/s13041-019-0468-x

**Published:** 2019-05-03

**Authors:** Jung-Hwa Tao-Cheng

**Affiliations:** NINDS Electron Microscopy Facility, National Institute of Neurological Disorders and Stroke, National Institutes of Health, Bethesda, MD 20892 USA

**Keywords:** Electron microscopy, PSD thickness, PSD curvature, Hippocampal slice cultures

## Abstract

Activity can induce structural changes in glutamatergic excitatory synapses, including increase in thickness and curvature of the postsynaptic density (PSD); these structural changes can only be documented by electron microscopy. Here in organotypic hippocampal slice cultures where experimental conditions can be easily manipulated, increases in thickness and curvature of PSDs were noticeable within 30 s of stimulation and progressed with time up to 3 min. These structural changes were reversible upon returning the samples to control medium for 5–10 min. Thus, the postsynaptic density is a very dynamic structure that undergoes rapid reorganization of its components upon stimulation, and recovery upon cessation of stimulation. The gradual increase in thickness of PSD could result from a gradual translocation of some PSD proteins to the PSD, and the increase in curvature of the PSD is likely led by postsynaptic elements.

**Electronic supplementary material:**

The online version of this article (10.1186/s13041-019-0468-x) contains supplementary material, which is available to authorized users.

## Introduction

The postsynaptic density (PSD) appears as dark material ~ 30 nm thick under the postsynaptic membrane in glutamatergic excitatory synapses of mammalian central nervous system by electron microscopy (EM). This easily-recognizable structural feature is illustrated in a classic atlas of neurocytology on various regions of exquisitely perfusion-fixed brains [1]. Subsequent contributions from immunogold labeling of various PSD proteins demonstrate that there is a second deeper layer of the PSD which is not always visible under basal conditions without special staining [2, 3]. Thus, the PSD can be subdivided into two layers: (1) the PSD core, a layer close to the postsynaptic membrane that is relatively stable either under basal or stimulated conditions, and (2) the PSD “pallium”, a deeper layer that is contiguous with the PD core, but extending further into the cytoplasm. It is this deeper layer of the PSD that becomes darker upon stimulation [3], and gives the appearance of PSD thickening.

Numerous EM studies on animal brains have shown PSDs of various thickness and curvature under different experimental conditions. However, much of this variability could be introduced by perfusion-fixation itself, which if carried out with a few minutes delay will cause hypoxia-induced excitatory stimulation, and result in increases of thickness and curvature of PSD [4]. Neuronal cell cultures offer a model system where the experimental conditions can be precisely controlled. Stimulation-induced thickening of PSD has been reported in dissociated hippocampal neuronal cultures [5–7], and in organotypic slice cultures of the hippocampus [8, 9]. However, these studies only compared PSDs under control basal conditions vs. those under intense excitatory stimulation.

The present study set out to further investigate the time course of structural changes of PSD in hippocampal organotypic slice cultures which maintain the in vivo stratifications of the hippocampus. The present study measured the thickness and curvature of the PSD upon depolarization and NMDA treatment as early as 30 s in order to capture early events. The time course and degree of the increases during stimulation and recovery upon secession of stimulation were documented.

## Methods

### Preparation, treatment and fixation of rat organotypic hippocampal slice cultures

All samples were from a previously published report [8] and reexamined here for changes in thickness and curvature of the PSD. Briefly, the hippocampus was removed from postnatal 6–8 day old rats and cut at 250 μm thickness with a tissue chopper. Slices were placed on cell culture inserts in six-well culture dishes and incubated 10–14 days in vitro before use. Culture dishes were placed on a floating platform in a water bath at 37 °C. Control incubation medium was HEPES-based Kreb’s Ringer at pH 7.4. High K^+^ medium was at 90 mM KCl, with osmolarity compensated by reducing the concentration of NaCl. N-methyl-D-aspartic acid (NMDA) medium incorporated 50 μM NMDA into the control medium. High K^+^ treatment was for 0.5, 1, 2, or 3 min, and NMDA treatment was for 0.5, 1, 2, or 5 min. To examine recovery after depolarization, high K^+^ medium was removed and the samples were washed three to four times in normal incubation medium for a total of 1, 5, and 10 min. Experimental controls were processed in parallel, including all the medium changes and washing steps. Slice cultures were fixed with 2% glutaraldehyde and 2% paraformaldehyde, or 4% glutaraldehyde in 0.1 N cacodylate buffer at pH 7.4 for 1–3 h at room temperature and then stored at 4 °C.

### Preparation, treatment and fixation of rat dissociated hippocampal neuronal cultures

All samples were from previously published reports [10] and used here to examine effects of different osmium treatment on the appearance of PSD thickness. Briefly, cell cultures were prepared from embryonic 20 day-old rat fetuses by papain dissociation, and then plated on glial feeder cultures, and experiments were carried out with three week-old cultures. Culture dishes were placed on a floating platform in a water bath maintained at 37 °C. Cell cultures were washed with control medium and treated for 2 min with either control or high K^+^ media, and then fixed immediately with 4% glutaraldehyde, or 2% glutaraldehyde and 2% paraformaldehyde in 0.1 N cacodylate buffer at pH 7.4 for 1–3 h at room temperature and then stored at 4 °C.

### Electron microscopy

All fixed slice cultures and most fixed dissociated cultures were washed in buffer and treated with 1% osmium tetroxide in 0.1 N cacodylate buffer at pH 7.4 for 1 h on ice. Some fixed dissociated cells samples were treated with “reduced osmium” (1% osmium tetroxide + 1% potassium ferrocyanide in cacodylate buffer at pH 7.4 on ice for 1 h) instead. Some additional samples were first treated with 1% tannic acid on ice for 1 h, followed by either 1% osmium tetroxide or “reduced osmium” for 1 h on ice. Samples were then washed and en bloc stained with 0.25–1% uranyl acetate in 0.1 N acetate buffer at pH 5.0 overnight at 4 °C, dehydrated with a series of graded ethanol, and finally embedded in epoxy resins. Thin sections were counterstained with uranyl acetate and lead citrate. Images were photographed with a bottom-mounted digital CCD camera (AMT XR-100, Danvers, MA, USA).

### Morphometry

#### Thickness and curvature of postsynaptic density

**Fig. 1:**
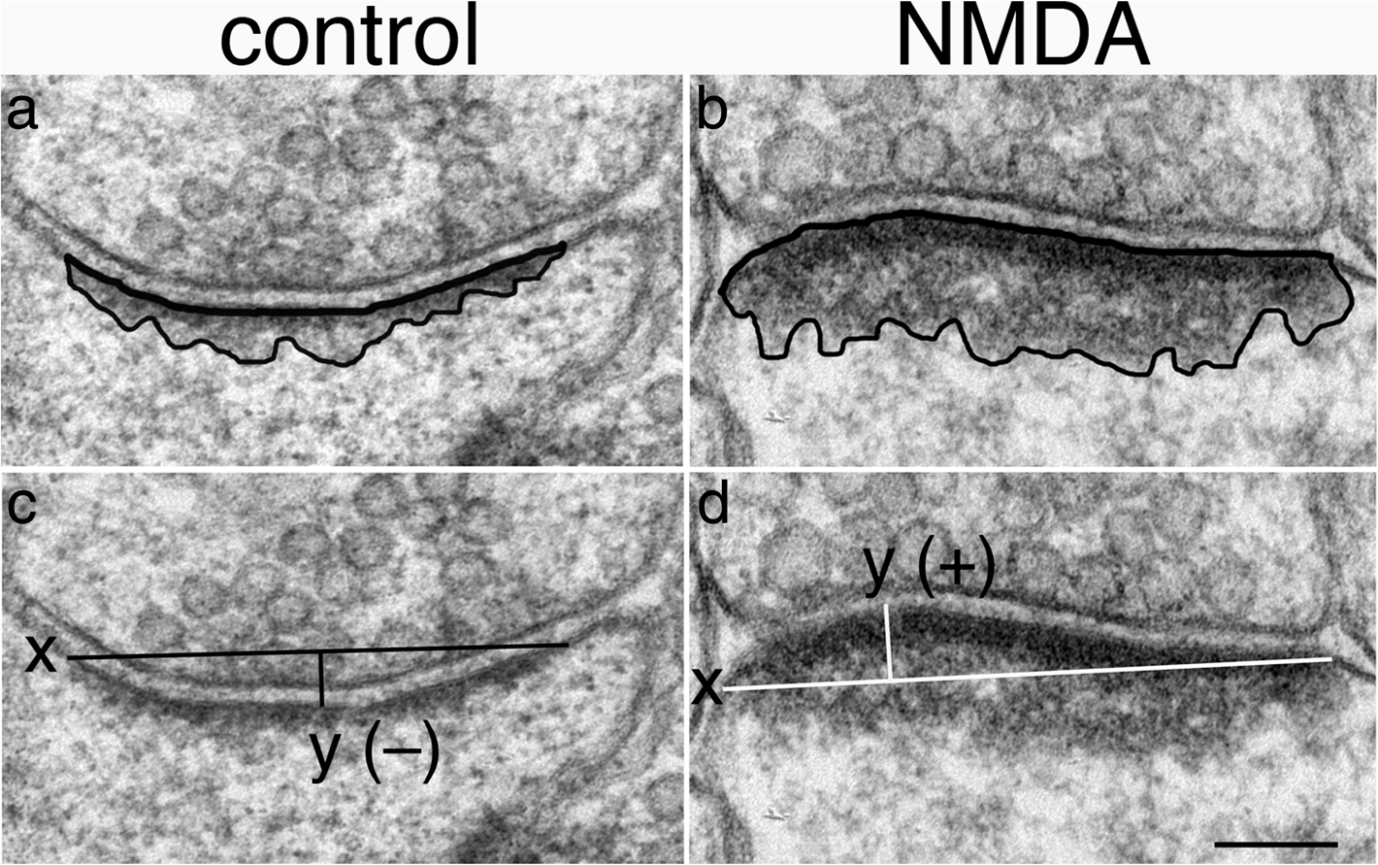
Methods of measurements of the average thickness of PSD (**a**, **b**) and the index value of curvature of PSD (c, d). Sample on left was treated with control media, and sample on right was treated with 50 μM of NMDA for 5 min. (**a**, **b**) –The area of the PSD was demarcated by the postsynaptic membrane and the border of the visible dark materials that were distinct from the rest of the cytoplasm. The area enclosed by these outlines was then divided by the length of the postsynaptic membrane to yield the “average thickness of the PSD”. (**c**, **d**) –The index for the curvature of the PSD was calculated by first drawing a line between the two ends of the postsynaptic membrane (the horizontal line indicated by x), and then a second line (the vertical line indicated by y) from the highest point of the arch of the postsynaptic membrane to the first line. The value of “y” divided by “x” times 100 is the index value for the curvature of the PSD. A negative value indicates that the presynaptic terminal arches into the postsynaptic spine (**c**), and a positive value indicates the reverse (**d**). Scale bar = 0.1 μm

Sampling of synapses from hippocampal slice cultures was restricted to stratum radiatum of the CA1 region immediately next to the clustered pyramidal neuronal somas, extending ~ 100 μm deep. This region covered approximately 5–7 grid openings in a 400-mesh honeycomb-patterned grid, and the entire area was photographed at 15,000x magnification. Every cross-sectioned excitatory synaptic profile with clearly delineated postsynaptic membrane was enlarged to 150,000x for measurement. On average, ~ 40 synaptic profiles were measured per sample (range, 27–51 synaptic profiles, Additional file 1). Methods for measuring thickness and curvature of PSDs were the same as described before [4]. Briefly, the average thickness of PSD was derived by marking the borders of the dark material underneath the postsynaptic membrane (Fig. 1, a, b) to measure the area of the PSD, and then divided by the length of the postsynaptic membrane. Area and length were measured with ImageJ (National Institutes of Health, Bethesda, MD, USA).

The index of curvature of PSD was calculated as stated in the figure legend of Fig. 1. Consistent with the designation used by Dyson and Jones [11], a negative value means that a presynaptic terminal arches into the PSD (Fig. 1c, also called a “smile” or “concave” curvature), and a positive value means that a PSD arches into the presynaptic terminal (Fig. 1d, also called a “frown” or “convex” curvature).

#### Statistical analysis

Comparisons between two groups were tested by Student’s t test. Comparisons among three groups or more were tested by one-way ANOVA with Tukey’s post-test. Data were presented as mean ± standard error of the mean (SEM).

## Results

### Stimulation increased the thickness and curvature of the postsynaptic density

**Fig. 2:**
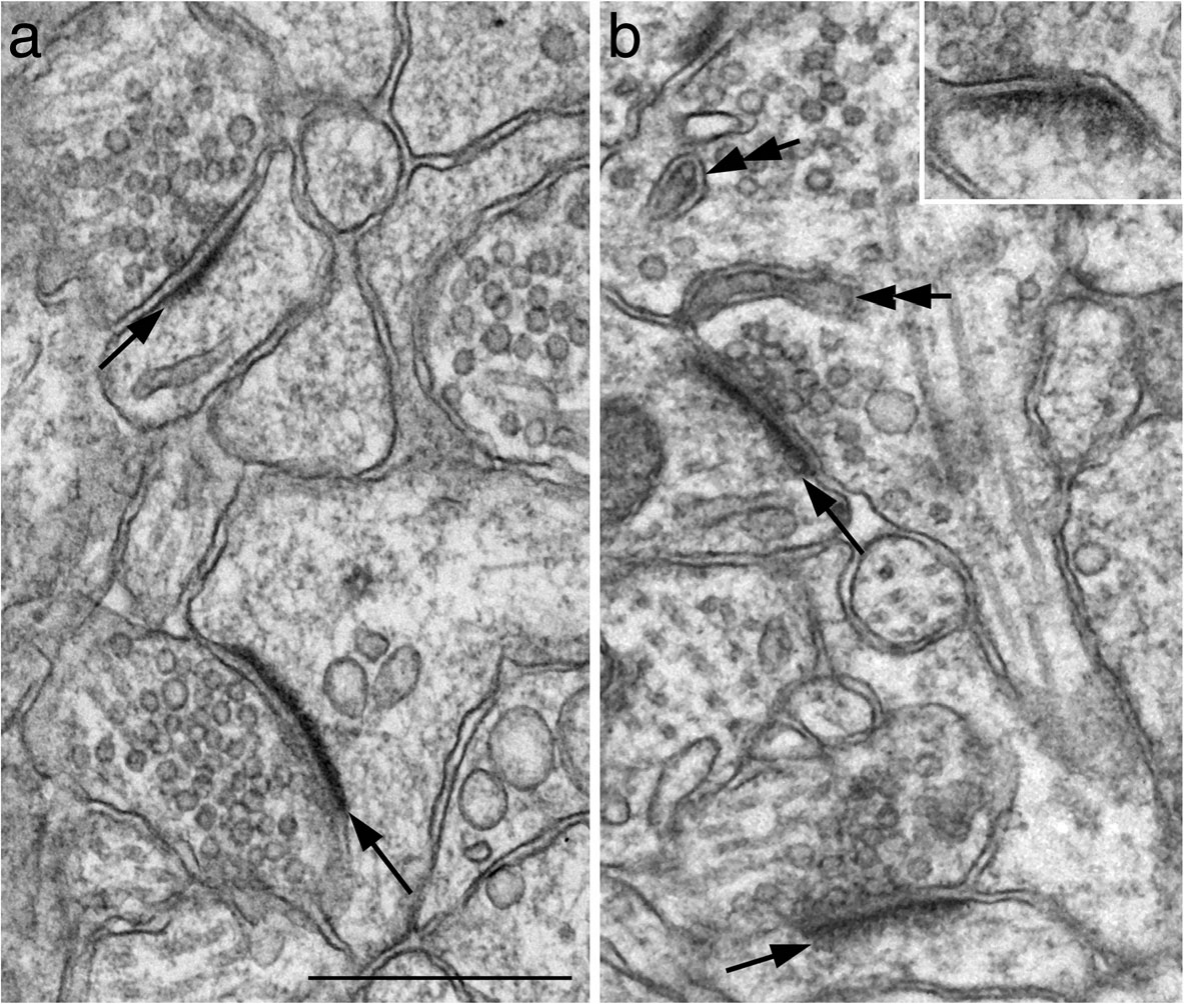
Synaptic profiles sampled from stratum radiatum of the CA1 region of hippocampal slice cultures under control conditions (**a**) and upon 30 s (**b**) and 2 min (inset in b) of depolarization with high K^+^ (90 mM). PSDs (arrows) showed a slight increase in thickness in b over a, and a more conspicuous thickness in inset in b over a. The curvature of the PSDs was in different directions in a vs. b. The presynaptic terminals arched into the postsynaptic elements slightly in a, while the reverse occurred in b. Double arrows in b points to double-membraned invaginations in the presynaptic terminals termed “spinules”, an activity-induced structure only seen in stimulated samples [9]. Scale bar = 0.5 μm

**Fig. 3:**
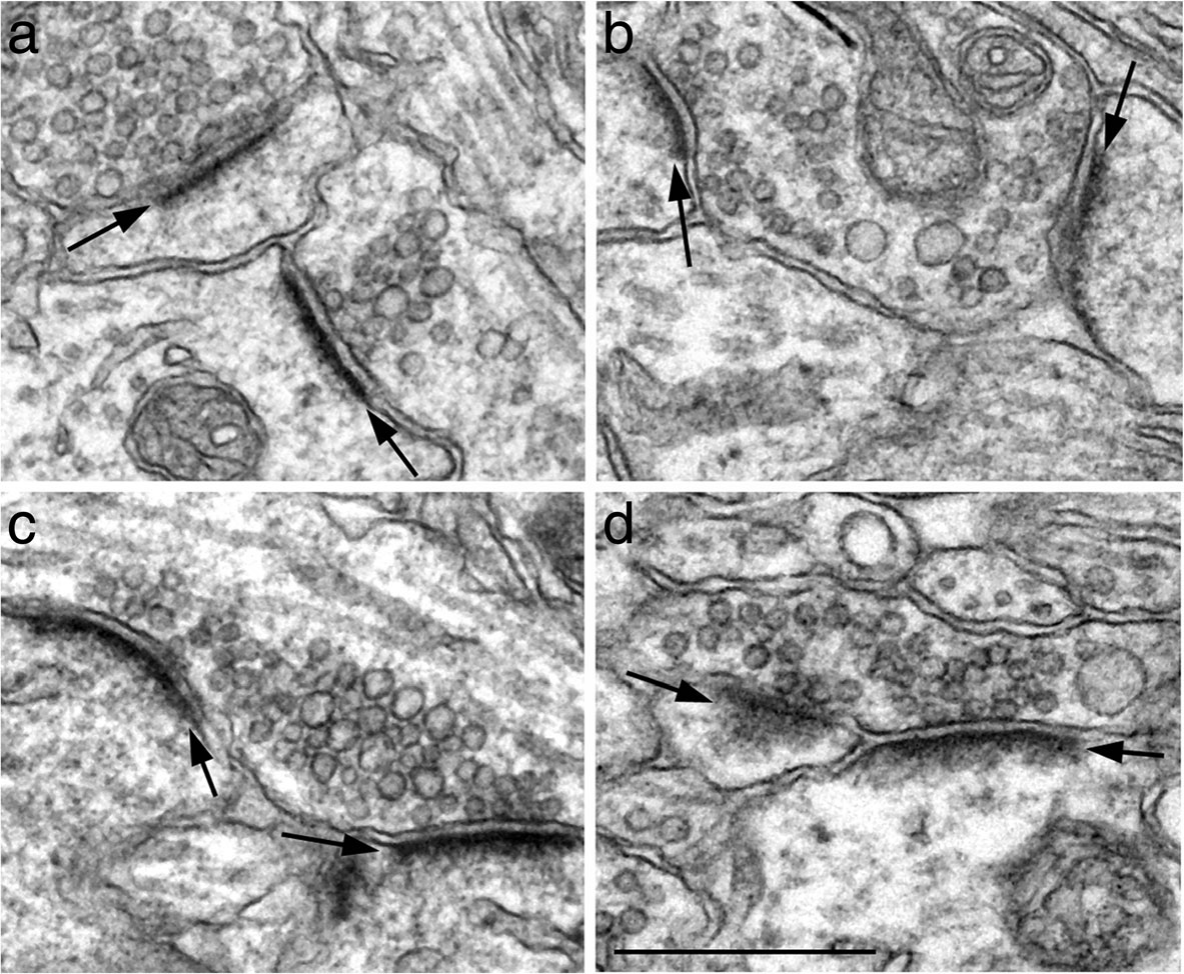
Synaptic profiles sampled from stratum radiatum of the CA1 region of the hippocampal slice cultures under control conditions (**a**) and upon 30 s (**b**), 1 min (**c**), and 2 min (**d**) of NMDA treatment (50 μM). PSDs (arrows) showed progressive increases in thickness from a to d. Typically, under control conditions (**a**), the curvature of PSDs was negative, but gradually increased into positive values (with the postsynaptic element arching into the presynaptic terminal) upon NMDA treatment (**b**, **c**, **d**). Scale bar = 0.5 μm

In control samples under basal conditions, the PSD appeared as a thin layer of dark material approximately 30–40 nm from the postsynaptic membrane (Fig. 1a). The deeper layer of the PSD (more than 40 nm from the postsynaptic membrane) was often less distinguishable, at least in staining intensity, from the adjacent cytoplasm (Fig. 1a, 2a, 3a). Most PSDs had a negative index value on the curvature, i. e., the presynaptic terminal arched into the postsynaptic dendrite (Fig. 1c, 2a, 3a). Upon stimulation, both the thickness and curvature of the PSD showed an increase within 30 s of depolarization with high K^+^ (Fig. 2) or NMDA treatment (Fig. 3), and these increases progressed with time.

### The thickness of PSD gradually increased with treatment time and this increase was reversible

**Fig. 4:**
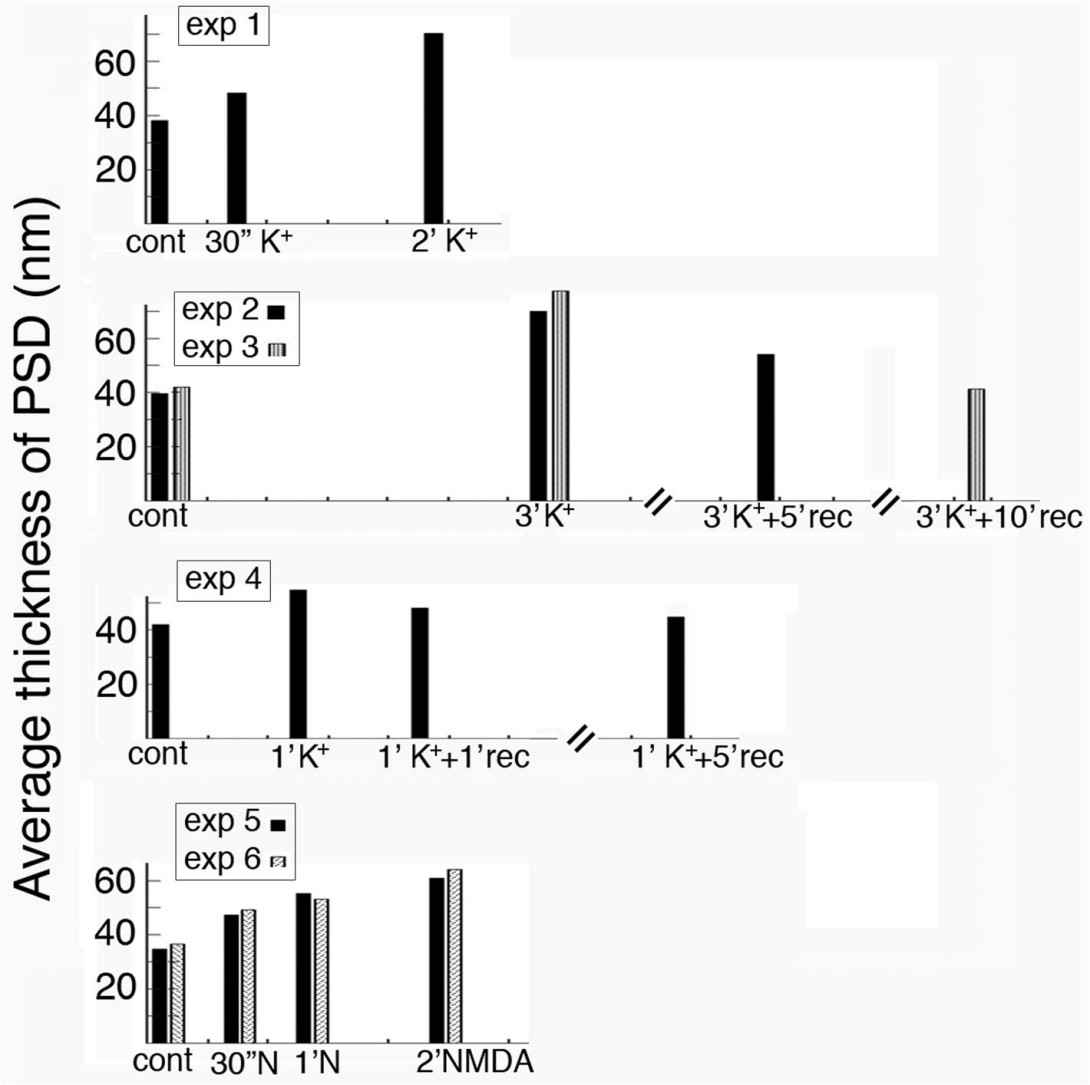
The average thickness of PSDs sampled from stratum radiatum of the CA1 region of hippocampal slice cultures consistently increased upon stimulation and decreased upon recovery. Complete data and statistical analyses are listed in Additional file 1

The average thickness of PSD from 6 control samples was 38.8 ± 1.2 nm (range 20–61, Additional file 1), a value significantly lower (*P* < 0.0001, Student’s t test) than that from 11 stimulated samples (59.1 ± 3.1 nm, range 30–116). Bar graphs in Fig. 4 show changes in average thickness of PSD from 6 experiments under different stimulation and recovery conditions. Mean values, sample sizes, ranges, and statistical analyses are listed in Additional file 1. The thickness of PSD gradually increased with treatment time as shown in experiment 1, 5, 6 in Fig. 4, and decreased upon wash-out of the high K^+^ medium, followed by recovery in control medium (exp 2, 3, 4 in Fig. 4). As expected, recovery was faster in exp. 4 with 1 min of depolarization than in exp. 2 & 3 with 3 min of depolarization.

**Fig. 5:**
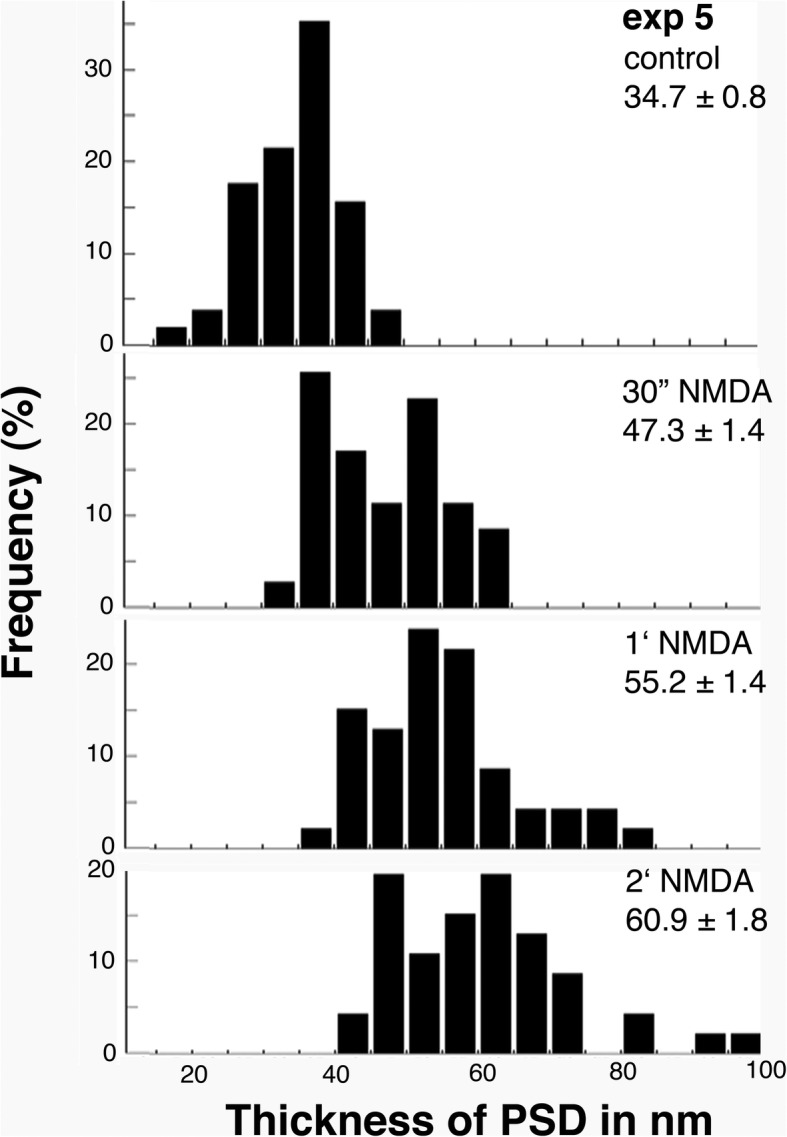
Histograms of thickness of PSD from exp. 5 in Additional file 1. Experimental conditions and average thickness of PSD are listed on the upper right corner of each panel. Both the range and mean values of average thickness of PSD gradually increased with NMDA treatment time. Statistical analyses are listed in footnotes of Additional file 1

The histograms in Fig. 5 show the thickness of PSD from one representative experiment. There was a progressive and significant increase in the thickness of PSD as the NMDA treatment time increased. Another figure of histograms showing a gradual decrease in thickness of PSD upon recovery is included as Additional file 2.

### The curvature of PSD gradually increased with treatment time and this increase was reversible

An index value of zero in the curvature of PSD means the PSD is flat, arching neither up or down. A negative value means the presynaptic terminal arches into the postsynaptic element (Fig. 1c), and a positive value means the postsynaptic element arches into the presynaptic terminal (Fig. 1d). The average index values for curvature of PSD from 6 control samples was − 1.2 ± 1.0 (ranged from − 4.1 to 2.6, Additional file 3), a value significantly lower (*P* < 0.0001, Student’s t test) than that from 11 stimulated samples (8.3 ± 1.1, ranged from 2.5 to 14.3).

**Fig. 6:**
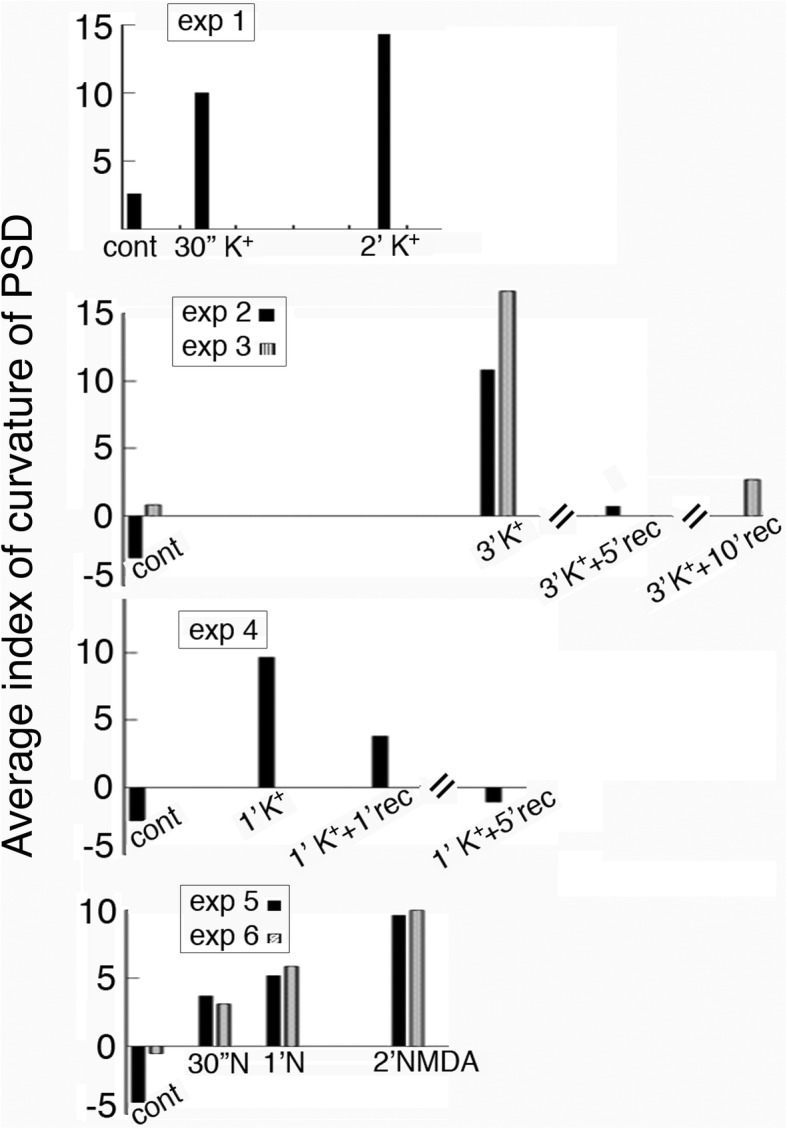
The curvature of PSD sampled from stratum radiatum of the CA1 region of hippocampal slice cultures consistently increased upon stimulation and decreased upon recovery. Complete data and statistical analyses are listed in Additional file 3

Bar graphs in Fig. 6 show changes in average index value on curvature of PSD from 6 experiments under different stimulation and recovery conditions. Mean values, ranges, and statistical analyses are listed in Additional file 3. The average index value for the curvature of PSD gradually increased with treatment time (exp 1, 5, 6 in Fig. 6), and decreased upon wash-out of the high K^+^ medium, followed by recovery in control medium (exp 2, 3, 4 in Fig. 6).

**Fig. 7:**
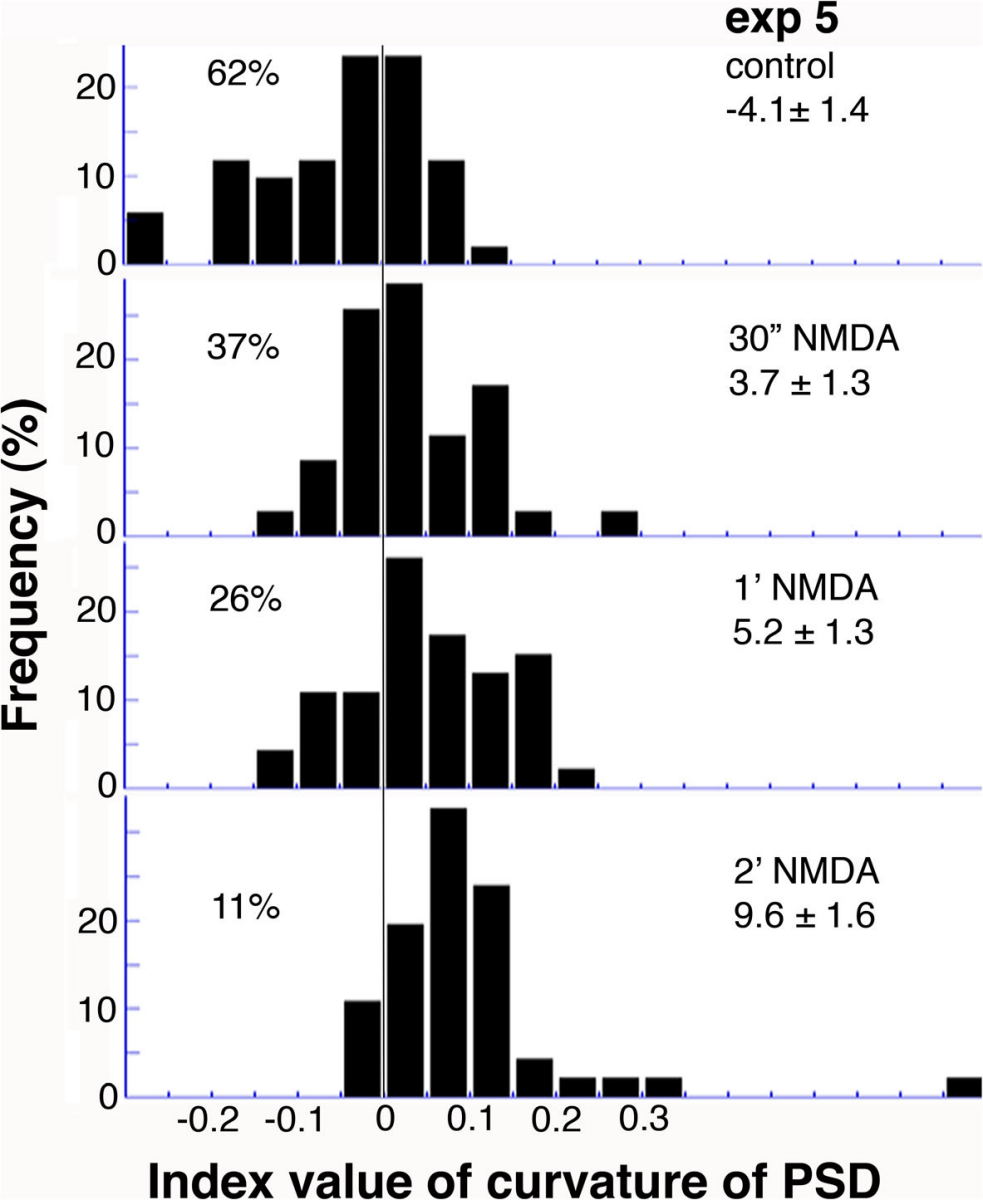
Histograms of index values of curvature of PSD from exp. 5 in Additional file 3. Experimental conditions and average index values are listed on the upper right corner of each panel. A vertical line divides the data points with negative values (including zeros), and the percent totals left of this line are listed on the upper left corner of each panel. Statistical analyses are listed in footnotes of Additional file 3

The histograms in Fig. 7 show the curvature of PSD from one representative experiment. As NMDA treatment time progressed, the distribution continued to shift toward a higher positive value, and the percent total of PSDs with negative index values (including zero’s) progressively decreased (Fig. 7). When pooling all experiments from Additional file 3 in calculating the percent total of PSDs with negative curvature, the average from 6 control samples was 55.5 ± 4.4%, a frequency significantly higher (*P* < 0.0001) than that from 11 stimulated samples (21.5 ± 3.5%). A second example showing a gradual decrease in curvature of PSD upon recovery is included as Additional file 4.

### Differential staining of PSD components by different osmium treatment

**Fig. 8:**
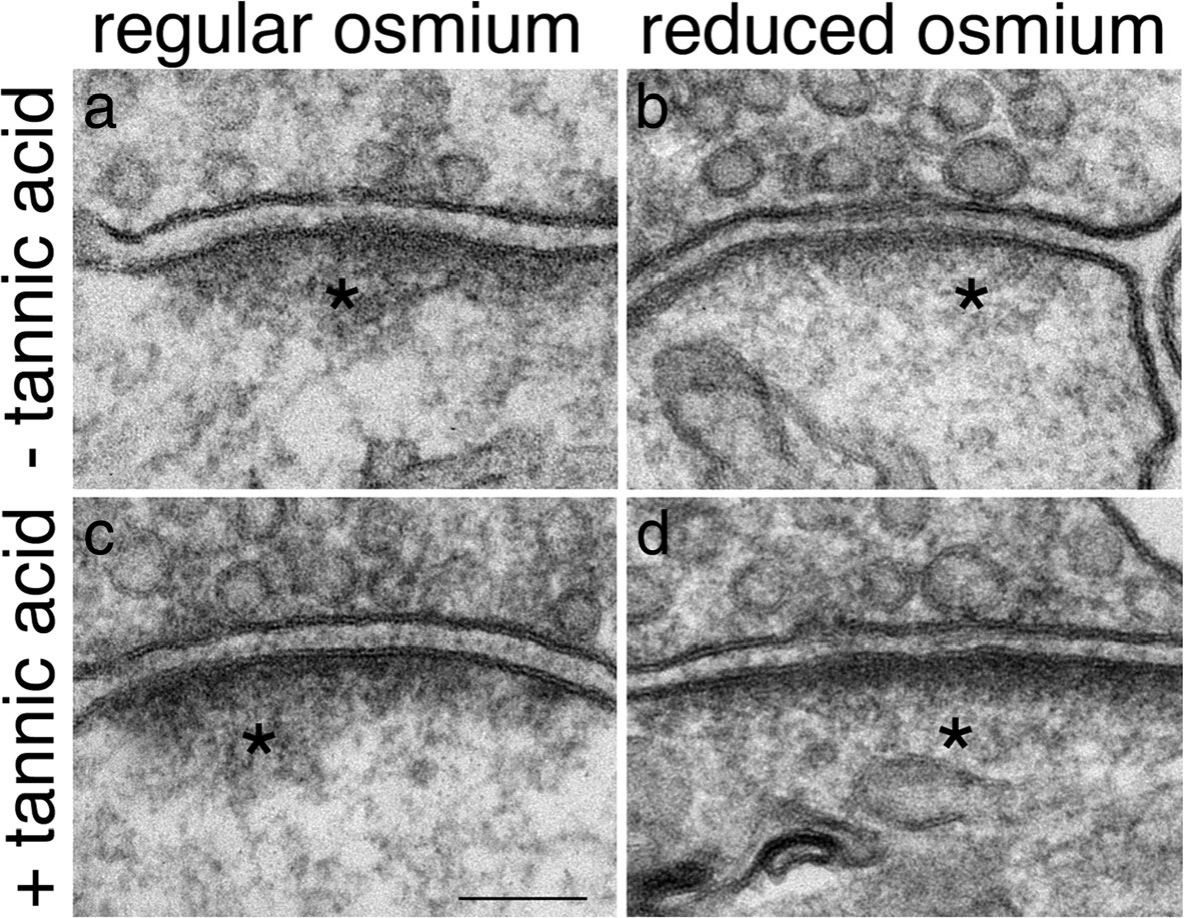
Four NMDA-treated (50 μM, 2 min) samples of dissociated hippocampal neuronal cultures were post-fixed differently as indicated. Samples were from sister cultures treated in parallel. The deeper layer of the PSD (marked by asterisks*) stained more prominently with regular osmium tetroxide (1% OsO_4_; a, c) than with “reduced osmium” (1% OsO_4_ and 1% potassium ferrocyanide; b, d). This preferential staining by regular osmium persisted with or without 1% tannic acid treatment. Scale bar = 0.1 μm

Interestingly, the stimulation-induced thickening of PSD was conspicuous when samples were treated with regular osmium (Fig. 8, left column), but much less so with “reduced osmium” (Fig. 8, right column). Thus, upon stimulation, some of the proteins aggregated at the deeper layer of the PSD [3] may be preferentially stained with regular osmium (asterisk in Fig. 8a) but less so with “reduced osmium” (asterisk in Fig. 8b). Adding tannic acid treatment prior to osmium treatment slightly increased the density of the PSD core (the dark layer of material within 30 nm of the postsynaptic density in Fig. 8d), but did not enhance the deeper layer of the PSD by much (asterisk in Fig. 8d).

## Discussion

The present study documented stimulation-induced increases in thickness and curvature of the PSD in the CA1 region of the hippocampus in slice cultures. This easily controllable experimental system offered the opportunity to capture initial structural changes as early as 30 s after stimulation, and to monitor the gradual increases as stimulation continues.

The PSD can be subdivided into two layers, a core that is ~ 30 nm thick proximal to the postsynaptic membrane, and a deeper layer (the pallium) extending further into the cytoplasm [3]. Notably, under control conditions, the deeper layer of PSD does not appear electron dense (Additional File 5a), although Homer and Shank are clearly present at this layer (Additional File 5b, c). Interestingly, upon stimulation, there is an increase in darkness of this deeper layer with “regular osmium” treatment but not with “reduced osmium” treatment. This technical issue should be taken into consideration when comparing data on “thickness of PSD” among different reports where samples may be treated with different osmium compositions.

The stimulation-induced increase in darkness of the deeper layer of the PSD can be attributed to additional proteins that translocate from the cytoplasm to the PSD [3]. These proteins include CaMKII, Shank, IRSp53, and CYLD. The most likely major contributor for the increased staining of this layer is CaMKII because of its great abundance at the PSD [12, 13] and its substantial increase (~ 3 fold) under excitatory conditions [6, 7], whereas Shank and IRSp53 increase only ~ 1.5 fold over controls [14–16]. CYLD, although greatly increased at the PSD upon stimulation [17], has an overall low abundance compared to these other PSD proteins [13].

Other candidates that could contribute to the increased electron density in this deeper layer include SynGAP and AIDA, two abundant proteins that move out of the PSD core upon stimulation, with a concomitant increase in the deeper layer of the PSD [18, 19]. In contrast, some proteins that are consistently located within the PSD core before or after stimulation cannot contribute to the increase in darkness of the deeper layer of the PSD. These proteins include PSD-95 [18], GKAP [15] and densin [20]. Other proteins like Homer [21] can also be ruled out because their concentrations in the deeper layer of the PSD do not change before or after stimulation.

The apparent gradual increase in PSD thickness with treatment time suggests that additional molecules may build up from close to the PSD core into the deeper layer of the PSD. For example, upon stimulation, CaMKII could initially translocate to near the PSD core to bind to NR2B [22] and/or densin [20], two proteins known to be highly concentrated at the PSD core. As stimulation progresses, additional CaMKII molecules can bind to those already translocated to the PSD core and continue to pile into thicker layers. Indeed, it is known that CaMKII molecules can self-aggregate [23, 24]. Alternatively, the additional CaMKII at the PSD brought on by stimulation might distribute evenly in the deeper layer of the PSD and bind to other scaffold proteins like Shank [25] that already exist in this layer. In this latter scenario, although CaMKII is not adding from PSD core outwards, other PSD proteins could still be added to the PSD in this fashion to account for the gradual increase in PSD thickness.

Change in curvature of the PSD under different conditions has been extensively studied [26–28], mostly in perfusion-fixed brains. There have been contradicting results as to whether neuronal activity increases or decreases the curvature of PSD. This contradiction could be due to sampling from different regions of the brain, different experimental protocols and/or morphometry methods, or simply due to the issue of perfusion-fixation itself that could have induced intense synaptic activity [4]. One way to bypass this potential complication caused by perfusion fixation is to immersion fix neuronal cultures whose synapses can be kept under basal conditions with certainty. The present study demonstrated that stimulation increases the curvature of PSD in hippocampal slice cultures, consistent with the finding from an earlier report in dissociated cortical neuronal cultures upon depolarization with high K^+^ [5].

What is the mechanism that caused the curvature of PSD to change? One immediate response to depolarization by high K^+^ or electrical stimulation is a massive release of synaptic vesicles from the presynaptic terminal [5, 29, 30]. The addition of membranes from fusion of these vesicles would increase the total area of presynaptic membrane [5, 30]. This increase could force the presynaptic terminal to wrap around the PSD and bend the PSD to arch into the presynaptic terminal yielding in a positive value in the curvature [26]. Alternatively, a postsynaptic mechanism might be involved. For example, upon stimulation, a change in actin configuration or interaction with PSD proteins [3, 26, 31] could exert a pull toward the core of the spine and result in a curvature change in the PSD. The present data support a postsynaptic mechanism, since NMDA treatment does not induce synaptic vesicle release, and yet it still increases the curvature of the PSD.

## Additional files

Additional file 1: Average (mean ± SEM in nm) thickness of PSD from excitatory synapses in stratum radiatum of the CA1 region of hippocampal slice cultures. (PDF 54 kb)

Additional file 2: Histograms of thickness of PSD. (PDF 2264 kb)

Additional file 3: Average (mean ± SEM) index of curvature of PSD from excitatory synapses in stratum radiatum of the CA1 region of hippocampal slice cultures. (PDF 53 kb)

Additional file 4: Histograms of index values of curvature of PSD. (PDF 2633 kb)

Additional file 5: Synaptic profiles from control samples under basal conditions. (PDF 1438 kb)
