# Supplementary material for: Stimulation induces gradual increases in the thickness and curvature of postsynaptic density of hippocampal CA1 neurons in slice cultures
Source: Mol Brain. 2019 May 3;12:44. doi: 10.1186/s13041-019-0468-x (PMC6499976; doi:10.1186/s13041-019-0468-x)
Supplement: Supplementary file 1 — Average (mean ± SEM in nm) thickness of PSD from excitatory synapses in stratum radiatum of the CA1 region of hippocampal slice cultures. (PDF 54 kb) [file 13041_2019_468_MOESM1_ESM.pdf]

Additional File 1. Average (mean ± SEM in nm) thickness of PSD from excitatory synapses in stratum radiatum of the CA1 region of hippocampal slice cultures.

**exp**	**control**	**30” K^+**	**1’ K^+**	**2’ K^+**	**3’ K^+**	**K^++ 1’ recovery**	**K^++ 5’ recovery**	**K^++ 10’ recovery**
**1**	38.1±1.1<br>(27)<br>[26-48]	48.1±1.3<br>(42)<br>[31-68]		70.2±2.4<br>(45)<br>[38-99]				
**2**	39.6±1.2<br>(28)<br>[30-51]				70.0±2.4<br>(38)<br>[42-94]		54.0±2.5<br>(47)<br>[28-115]	
**3**	41.9±1.1<br>(45)<br>[30-61]				77.5±1.6<br>(27)<br>[58-116]			41.2±0.9<br>(35)<br>[27-54]
**4**	42.0±1.3<br>(40)<br>[24-61]		54.8±1.6<br>(46)<br>[30-76]			48.0±1.5<br>(48)<br>[27-74]	44.8±1.5<br>(51)<br>[22-81]	
**Mean± SEM**	40.4±0.9	51.5±3.4		72.6±2.5		47.0±2.7		

	**control**	**30” NMDA**	**1’ NMDA**	**2’ NMDA**
**5**	34.7±0.8<br>(51)<br>[20-47]	47.3±1.4<br>(35)<br>[34-64]	55.2±1.4<br>(46)<br>[39-81]	60.9±1.8<br>(46)<br>[44-96]
**6**	36.5±0.9<br>(38)<br>[27-47]	49.1±1.2<br>(42)<br>[38-68]	53.1±1.9<br>(36)<br>[32-85]	64.0±2.1<br>(34)<br>[46-95]
**Mean± SEM**	35.6±0.9	48.2±0.9	54.2±1.1	62.5±1.2

(n) = number of synaptic profiles measured.

[range] = minimum – maximum values.

ANOVA with Tukey’s pairs comparison:

Exp 1: control vs. 30” K^+ ($P<0.005$); cont vs. 2’ K^+ ($P<0.0001$); 30” vs. 2’ K^+ ($P<0.0001$).

Exp 2: $P<0.0001$ for all 3 pairs of comparison.

Exp 3: 3’ K^+ highly significant ($P<0.0001$) from control and 3’ K^+ + 10’ recovery.

Exp 4: control vs. 1’K^+ ($P<0.0001$); cont. vs. 1’K^+ + 1’ recovery ($P<0.05$); 1’K^+ vs. 1’K^+ + 1’ recovery ($P<0.01$); 1’K^+ vs. 1’K^+ + 5’recovery ($P<0.0001$).

Exp 5: control highly significant ($P<0.0001$) from all 3 NMDA samples. 30” NMDA vs. 1’ NMDA ($P<0.005$); 30” NMDA vs. 2’ NMDA ($P<0.0001$); 1’ NMDA vs. 2’ NMDA ($P<0.05$).

Exp 6: except for no significance between 30” and 1’ NMDA, all other pairs-comparison highly significant ($P<0.0001$).
