# Supplementary material for: Stimulation induces gradual increases in the thickness and curvature of postsynaptic density of hippocampal CA1 neurons in slice cultures
Source: Mol Brain. 2019 May 3;12:44. doi: 10.1186/s13041-019-0468-x (PMC6499976; doi:10.1186/s13041-019-0468-x)
Supplement: Supplementary file 2 — Histograms of thickness of PSD. (PDF 2264 kb) [file 13041_2019_468_MOESM2_ESM.pdf]

Additional File 2. Histograms of thickness of PSD.

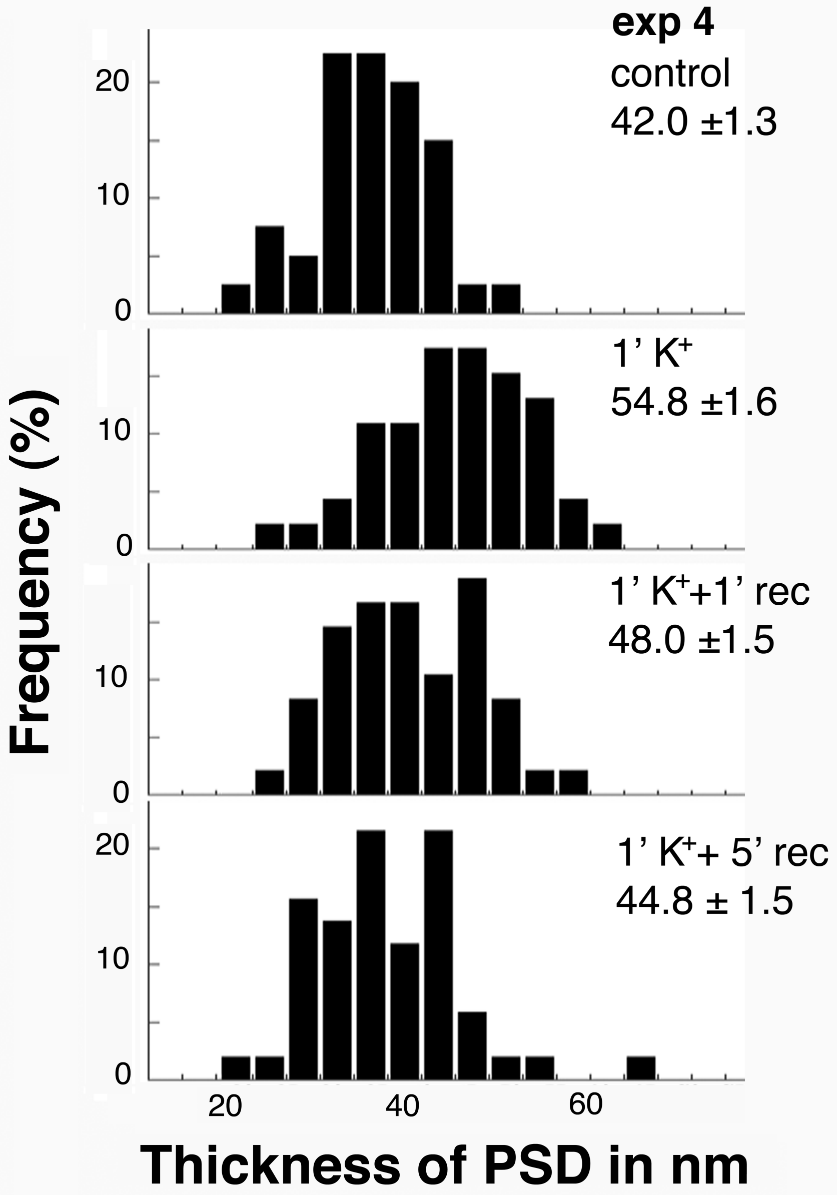

Experimental conditions and average thickness are listed on the upper right corner of each panel. Statistical analyses are listed in footnotes of Additional File 1. There is a significant increase in PSD thickness upon 1' depolarization with high K^+, and a gradual decrease upon recovery (rec) for 1' and 5' minutes.
