# Supplementary material for: Stimulation induces gradual increases in the thickness and curvature of postsynaptic density of hippocampal CA1 neurons in slice cultures
Source: Mol Brain. 2019 May 3;12:44. doi: 10.1186/s13041-019-0468-x (PMC6499976; doi:10.1186/s13041-019-0468-x)
Supplement: Supplementary file 3 — Average (mean ± SEM) index of curvature of PSD from excitatory synapses in stratum radiatum of the CA1 region of hippocampal slice cultures. (PDF 53 kb) [file 13041_2019_468_MOESM3_ESM.pdf]

**Additional File 3. Average (mean ± SEM) index of curvature of PSD from excitatory synapses in stratum radiatum of the CA1 region of hippocampal slice cultures.**

| exp | control | 30” $K^+$ | 1’ $K^+$ | 2’ $K^+$ | 3’ $K^+$ | $K^+$+ 1’ recovery | $K^+$+ 5’ recovery | $K^+$+ 10’ recovery |
|---|---|---|---|---|---|---|---|---|
| **1** | 2.6±1.2 [-13, 14] | 10.0±1.2 [-8, 31] | | 14.3±2.1 [-6, 67] | | | | |
| **2** | -3.1±1.6 [-23, 13] | | | | 10.8±2.1 [-18, 45] | | 0.7±1.4 [-23, 28] | |
| **3** | 0.6±1.2 [-24, 13] | | | | 12.5±1.6 [-12, 36] | | | 2.0±0.9 [-13, 28] |
| **4** | -2.5±1.6 [-27, 11] | | 9.6±1.3 [-9, 36] | | | 3.8±1.3 [-22, 28] | -1.1±0.9 [-16, 21] | |
| **Mean± SEM** | -0.6±1.4 | 9.8±0.2 | | 12.5±1.0 | | 1.4±1.0 | | |

| exp | control | 30” NMDA | 1’ NMDA | 2’ NMDA |
|---|---|---|---|---|
| **5** | -4.1±1.4 [-30, 13] | 3.7±1.3 [-14, 25] | 5.2±1.3 [-11, 24] | 9.6±1.6 [-2, 66] |
| **6** | -0.4±1.1 [-20, 10] | 2.5±1.2 [-23, 19] | 4.7±1.0 [-5, 21] | 8.0±1.2 [-6, 24] |
| **Mean± SEM** | -2.3±1.9 | 3.1±0.6 | 5.0±0.3 | 8.8±0.8 |

Experiment numbers and sample sizes (n) are the same as in Additional File 1.

[range] = minimum and maximum values.

ANOVA with Tukey’s post comparison:

Exp 1: control vs. 30” $K^+$ ($P<0.0001$); control vs. 2’ $K^+$ ($P<0.0001$).

Exp 2: control vs. 3’ $K^+$ ($P<0.0001$); 3’ K vs. 3’ K + 5’ recovery ($P<0.0001$).

Exp 3: control vs. 3’ $K^+$ ($P<0.0001$); 3’ K vs. 3’ K + 10’ recovery ($P<0.0001$).

Exp 4: control vs. 1’ $K^+$ ($P<0.0001$); control vs. 1’ K + 1’ recovery ($P<0.005$); 1’ K vs. 1’ K + 1’ recovery ($P<0.01$); 1’ K vs. 1’ K + 5’ recovery ($P<0.0001$); 1’ K + 1’ recovery vs. 1’ K + 5’ recovery ($P<0.05$).

Exp 5: control vs. 30” NMDA ($P<0.005$); control vs. 1’ NMDA ($P<0.0001$); control vs. 2’ NMDA ($P<0.0001$); 30” NMDA vs. 2’ NMDA ($P<0.05$).

Exp 6: control vs. 1’ NMDA ($P<0.05$); control vs. 2’ NMDA ($P<0.0001$); 30” NMDA vs. 2’ NMDA ($P<0.005$).
