# Supplementary material for: Stimulation induces gradual increases in the thickness and curvature of postsynaptic density of hippocampal CA1 neurons in slice cultures
Source: Mol Brain. 2019 May 3;12:44. doi: 10.1186/s13041-019-0468-x (PMC6499976; doi:10.1186/s13041-019-0468-x)
Supplement: Supplementary file 4 — Histograms of index values of curvature of PSD. (PDF 2633 kb) [file 13041_2019_468_MOESM4_ESM.pdf]

Additional File 4. Histograms of index values of curvature of PSD.

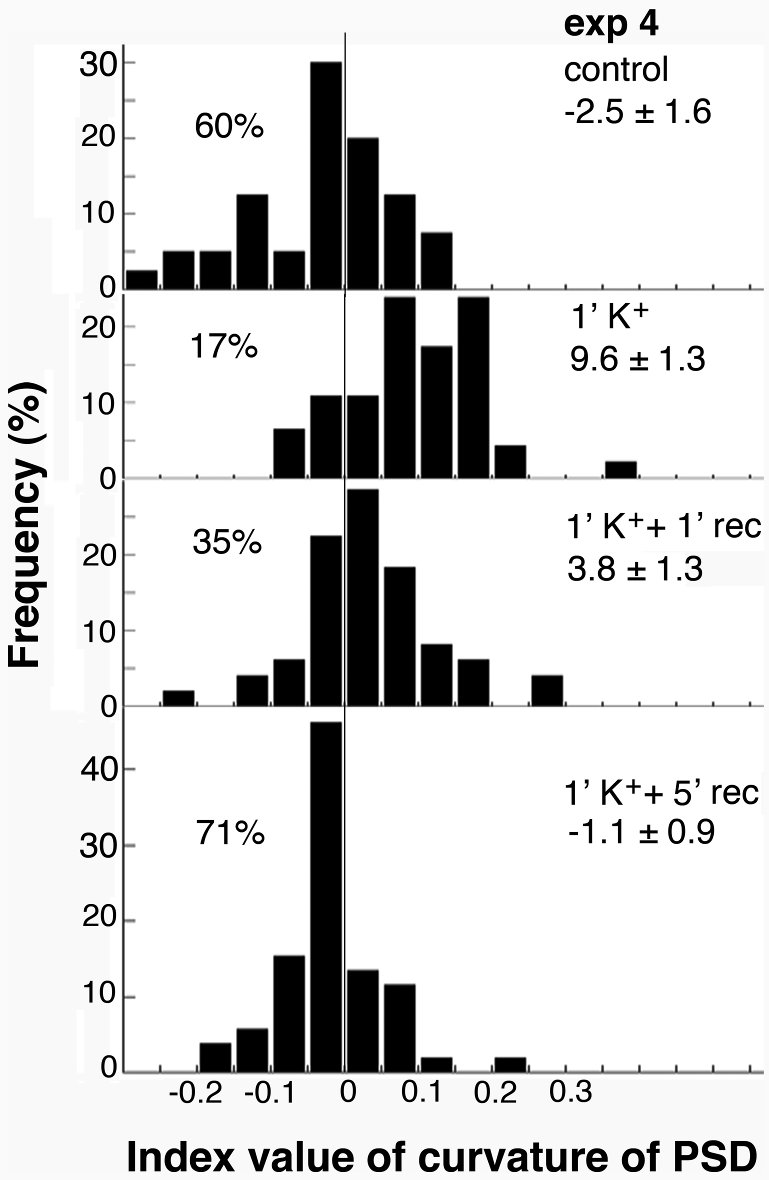

Experimental conditions and average index values of curvature are listed on the upper right corner of each panel. A vertical line divides the data points with negative values (including zeros), and the percent totals left of this line are listed on the upper left corner of each panel. Statistical analyses are listed in footnotes of Additional File 3. There was a significant increase in curvature index upon 1' depolarization with high K^+, and a gradual decrease upon recovery (rec) for 1' and 5' minutes.
