# Supplementary material for: Stimulation induces gradual increases in the thickness and curvature of postsynaptic density of hippocampal CA1 neurons in slice cultures
Source: Mol Brain. 2019 May 3;12:44. doi: 10.1186/s13041-019-0468-x (PMC6499976; doi:10.1186/s13041-019-0468-x)
Supplement: Supplementary file 5 — Synaptic profiles from control samples under basal conditions. (PDF 1438 kb) [file 13041_2019_468_MOESM5_ESM.pdf]

Additional File 5. Synaptic profiles from control samples under basal conditions.

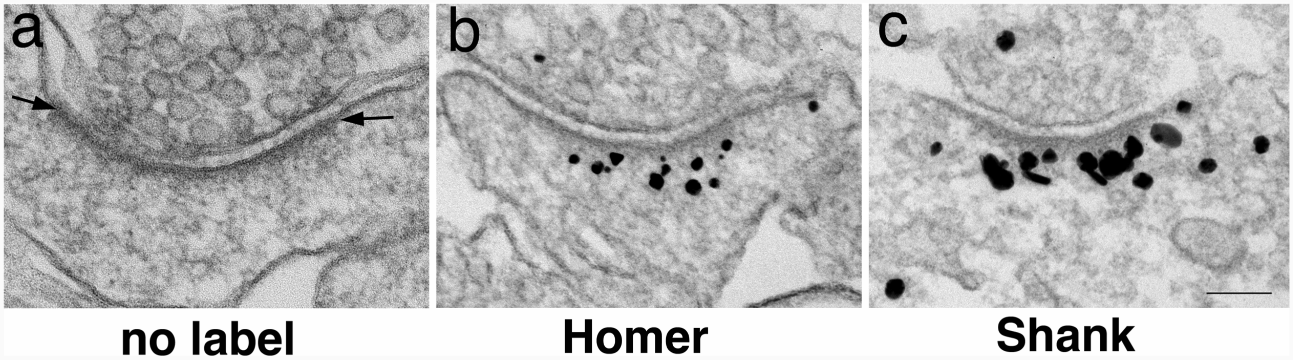

(a) A typical synaptic profile without any immunolabeling. The PSD (area between two arrows) appeared as a thin layer (~ 30 nm) of electron dense material underneath the postsynaptic membrane. (b, c) Immunogold labeling of Homer (b) and Shank (c) are distinctly localized to the deeper layer of the PSD, beyond the PSD core. This layer is not readily detectable without immunolabeling. Scale bar = 0.1 μm.
